# The Biomarkers for Acute Myocardial Infarction and Heart Failure

**DOI:** 10.1155/2020/2018035

**Published:** 2020-01-16

**Authors:** Xi-Ying Wang, Fen Zhang, Chi Zhang, Liang-Rong Zheng, Jian Yang

**Affiliations:** Department of Cardiology, The First Affiliated Hospital, College of Medicine, Zhejiang University, Hangzhou, China

## Abstract

The use of a large number of cardiovascular biomarkers, meant to complement the use of the electrocardiogram, echocardiography cardiac imaging, and clinical symptom assessment, has become a routine in clinical diagnosis, differential diagnosis, risk stratification, and prognosis and guides the management of patients with suspected cardiovascular diseases. There is a broad consensus that cardiac troponin and natriuretic peptides are the preferred biomarkers in clinical practice for the diagnosis of the acute coronary syndrome and heart failure, respectively, while the roles and possible clinical applications of several other potential biomarkers are still under study. This review mainly focuses on the recent studies of the roles and clinical applications of troponin and natriuretic peptides, which seem to be the best-validated markers in distinguishing and predicting the future cardiac events of patients with suspected cardiovascular diseases. Additionally, the review briefly discusses some of the large number of potential markers that may play a more prominent role in the future.

## 1. Introduction

According to the World Health Organization (WHO), cardiovascular disease (CVD) is the number one cause of death globally and is responsible for 45% of all deaths, equating to >4 million deaths per year in Europe [1]. Its high morbidity, mortality, and high rate of rehospitalization have forced a number of researchers to search for the best way to diagnose, stratify risk, and manage patients with suspected cardiovascular diseases, among which acute coronary syndrome (ACS) and heart failure (HF) are most commonly studied. Acute coronary syndrome, usually caused by decreased coronary artery perfusion due to stenosis or distal embolization of the thrombus [2] and sudden total occlusion of a coronary artery by thrombosis, typically presents with the main symptoms of acute chest pain and persistent ST-segment elevation in an electrocardiogram (ECG). However, there are a small number of patients without obvious symptoms or changes in the ECG. Thus, the measurement of a number of cardiac biomarkers is urgently needed to help with early diagnosis, risk stratification, and management of acute coronary syndrome. Heart failure is the terminal stage of a wide range of cardiovascular diseases that result in the decompensation of the heart's ability to contract or relax, also defined as a clinical condition with typical symptoms and signs [3]. The pathophysiological process involves the release of a series of factors, hormones, and proteins into the bloodstream, which could subsequently be used as diagnostic biomarkers.

Classically, biomarkers, such as cardiac troponin and the natriuretic peptides (NPs), associated with acute coronary syndromes and heart failure, respectively, play an important role in routine clinical practice. The ideal biomarker for detecting myocardial injury needs to be expressed at relatively high levels within cardiac tissue, with high clinical sensitivity and specificity that is detectable in the blood early after the onset of symptoms, such as chest pain [4]. As there are numerous cardiac biomarkers, it is useful to classify biomarkers into various pathophysiologic groups, such as myocardial ischemia or necrosis, inflammation, hemodynamics, angiogenesis, atherosclerosis, or plaque instability [5]. Cardiac troponin (cTn), expressed as three similar isoforms (I, C, and T), is the biomarker of choice for the diagnosis of myocardial necrosis because it is the most sensitive and specific biochemical marker of myocardial ischemia/necrosis available [6]. It has been demonstrated that plasma cTn content is elevated in many cardiovascular diseases other than acute myocardial infarction, including acute or chronic heart failure, aortic dissection, myocarditis, takotsubo cardiomyopathy, atrial fibrillation, and stroke [7]. The mechanisms underlying the release of cTn into the bloodstream are believed to include cell turnover, myocyte apoptosis, necrosis and reversible injury, increased cell membrane permeability, and release of cardiac troponin degeneration products [7, 8]. Studies have also shown that membranous blebs enable the release of cardiac troponin in response to ischemia without necrosis [9]. Among the isoforms, the most specific markers for acute coronary syndromes are cardiac troponin I (cTnI) and cardiac troponin T (cTnT), the elevations of which have become a predominant indicator for acute myocardial infarction (AMI) [10] and are considered the “gold standard” in AMI diagnosis.

Natriuretic peptides (NPs), composed of three structurally similar peptides, that is, atrial natriuretic peptide (ANP), B-type (or brain) natriuretic peptide (BNP), and C-type natriuretic peptide (CNP), play an important role in cardiovascular disease [11] and are elevated to a large extent in response to increased wall stretching due to volume or load stress in HF. BNPs play a critical role in maintaining homeostasis in the cardiovascular system, serving as counterregulatory hormones for volume and pressure overload [12]. The levels of blood BNP and the N-terminal pro-B-type natriuretic peptide (NT-proBNP) are widely measured in clinical applications for the diagnosis, risk stratification, and management of patients with heart failure, as they are the closest to optimal biomarker standards for clinical implications in HF [13]. Higher natriuretic peptide levels increase the likelihood that the etiology of dyspnea is due to HF [14].

We performed PubMed and Web of Science searches for original studies and screened reference lists to identify possible relevant studies by searching the literature from 2003 to 2019. Search terms were “biomarker,” “new biomarker,” “B-type natriuretic peptide,” “cardiac troponin,” “C-reactive protein,” “copeptin,” “endothelial cell-specific molecule 1,” “interleukin-6,” “inflammatory medium,” “cardiac myosin-binding protein C,” “gene expression biomarkers,” “heart-type fatty acid binding protein,” “platelet related biomarkers,” “transmembrane and soluble isoforms,” “cystatin C,” “microRNA,” “acute coronary disease,” “coronary artery disease,” “heart failure,” “non–ST-elevation acute coronary syndrome,” etc. Studies were included if they were prospective, retrospective, randomized controlled trials or animal models.

This article will provide an overview of cardiac biomarkers, mainly focusing on the recent studies of the role and applications of cTn and NPs in the diagnosis, risk stratification, and management of patients with acute coronary syndromes and heart failure. Additionally, this article reviews some biomarkers related to acute coronary syndromes and heart failure that may have potential clinical value in the future.

## 2. The Most Popular and Effective Biomarkers

### 2.1. Cardiac Troponin

The cTn complex is one of the components of the thin filament and it plays a significant role in the regulation of muscle contraction. This complex is composed of three isoforms, namely, cTnC, which binds Ca^2+^, cTnI, which inhibits the ATPase activity of actomyosin, and cTnT, which interacts with actomyosin. cTn mediates the interaction between actin and myosin and thereby regulates cardiomyocyte contraction [15]. cTnI and cTnT are the two isoforms expressed in the cardiac muscle only (cTnC is also expressed in the skeletal muscle), and they have been verified to be specific and sensitive biomarkers of myocardial damage [16–18], which is particularly important in asymptomatic patients, when combined with other biomarkers and examinations [19]. Many researches have proved that cTn is a more sensitive and specific marker of cardiomyocyte injury than creatine kinase (CK), its MB isoenzyme (CK-MB), and myoglobin [17]. In clinical practice, high-sensitivity cTn (hs-cTn) assays are recommended over sensitive cTn or traditional cTn [16–18, 20] because they enable accurate quantification of troponin in most healthy people [21]. In addition, the hs-cTn assay presents a superior prognostic performance in the non–ST-elevation acute coronary syndrome (NSTE-ACS), compared to the commercial fourth-generation cTnT assay [22, 23].

cTn is an integral criterion in the diagnosis of AMI. According to the 2015 European Society of Cardiology (ESC) guidelines for NSTE-ACS, the measurement of a biomarker of cardiomyocyte injury, preferably hs-cTn, was mandatory in all patients with suspected NSTE-ACS [20]. This can serve to confirm the diagnosis in symptomatic patients with diagnostic electrocardiographic changes [24]. An undetectable level of hs-cTn at presentation has a high negative predictive value and allows the rapid rule-out of AMI in patients with acute chest pain [25]. The elevation of hs-cTn usually occurs within 3–12 hours and persists for 5–14 days after the onset of symptoms in patients with AMI [4]. Data from several large multicenter studies have consistently shown that hs-cTn assays increased the accuracy of the AMI diagnosis at the time of presentation to the emergency department (ED) [16, 20, 26]. Blood levels of hs-cTn must be evaluated at the time when patients come into ED with the complaint of chest pain. If the level is over the upper limit of normal (ULN) or the pain persists no more than 6 hours, especially without typical changes in ECG, hs-cTn should be retested [20]. When the initial level is above ULN or the retest level is increased accompanied by typical changes in the ECG, the patient should be treated as soon as possible because the diagnosis of AMI is highly suspected. However, if the initial level of hs-cTn is normal, the time interval to the second cardiac troponin assessment remains controversial. For rapid rule-out in AMI patients with hs-cTn, two alternative approaches using the 0 h/1 h algorithm or 0 h/3 h algorithm have been adequately validated and may be considered [20]. It has been confirmed that the addition of the 1h-hs-cTn measurement significantly promotes the diagnostic accuracy in the patients with mild cTn elevation [27]. The ESC recommended that the rule-in using the 0 h/3 h algorithm has a high positive predictive value, while the sensitivity remains too low for clinical use [28]. However, increasing age reduces the rule-in accuracy of the 0 h/1 h algorithm, and therefore, a modest increase in the cut-off point is recommended which can still maintain the rule-out safety [29]. With regard to the gender factor, researches have demonstrated that the gender-specific hs-cTnT cut-off point only has modest influence compared to the age factor [30]. Another study showed that the downward adjustment of hs-cTn thresholds in women may be warranted to reduce the underdiagnosis of acute myocardial infarction in women [31]. It was also confirmed that revised cut-offs for hs-cTnT based on age and gender only improve the diagnostic performance rather than the prognostic risk prediction for death or other adverse events [32].

One report has shown that while hs-cTnT and hs-cTnI seem to have comparable diagnostic accuracies, hs-cTnI had greater early diagnostic accuracy [33]. That suggests that hs-cTnI can be examined separately to rule-in or rule-out patients in time if possible in the advanced analytical technique. Additionally, another study showed that the hs-cTnT blood concentration exhibited a diurnal rhythm, characterized by gradually decreasing concentrations throughout the daytime, rising concentrations during nighttime, and peak concentrations in the morning [34]. The rhythm does not seem to affect the diagnostic accuracy for AMI, except for screening purposes. hs-TnI does not seem to express the same rhythm, it demonstrates high diagnostic accuracy for AMI, and it does not differ with time of presentation [35].

Beyond diagnostic utility, cTn levels provide prognostic information in predicting short- and long-term outcomes based on clinical and ECG variables. Numerous studies have demonstrated a strong independent relationship between cTn and prognosis [36]. Meta-analysis indicated that in patients with NSTE-ACS the short-term odds of death were increased three- to eightfold for patients with an abnormal troponin test, and in patients with suspected ACS, those who had a negative troponin test had an overall mortality between 0.7% (troponin I, cohort studies) and 2.1% (troponin I, trial studies) [36]. However, unlike in the diagnosis of AMI, frequent and serial measurements are not necessary, because an isolated measurement on the first postoperative day is enough to identify high-risk subgroups, and changes of hs-cTn do not seem to further improve risk stratification beyond initial presentation values [26]. In the study by Shah et al., almost two-thirds of the patients with suspected ACS could have been discharged with very few cardiac events, using the standard of cTn concentration of less than 5 ng/L [21]. However, for those who are well within the normal reference interval, increasing cTn concentrations is positively associated with adverse cardiovascular outcomes in primary prevention populations [37]. Both hs-cTnI and hs-cTnT were predictive for all-cause mortality. Notably, hs-cTnT measurement showed superior prognostic performance in predicting long-term all-cause mortality compared with hs-cTnI [38].

### 2.2. BNP or NT-proBNP

BNP is synthesized and released by cardiac ventricular cells in response to volume or pressure overload [39]. Both active BNP and inactive NT-proBNP are generated from the cleavage of proBNP and therefore they are secreted into the bloodstream in equal concentrations [40]. While ANP is stored as the preform in the intracellular granules, BNP is predominantly synthesized when triggered by extracellular stimuli. After secretion into the bloodstream, the BNP will then bind to NP receptors (NPRs) and subsequently activate the intracellular cGMP signaling cascades to reduce the volume or pressure overload. BNP is primarily cleared through the degradation by neutral endopeptidases and partially through the uptake by NPR and renal excretion [41]. BNP and NT-proBNP, the two most commonly used natriuretic peptides, play a diagnostic role in the assessment of heart failure [42]. They may be increased due to systolic and/or diastolic dysfunction, left ventricular hypertrophy, valvular heart disease, ischemia, or a combination of these factors [43]. In multiple logistic-regression analyses, the measurements of B-type natriuretic peptide added significant independent predictive power to other clinical variables in models predicting which patients had congestive heart failure, with an odds ratio of 29.60. It is suggested that BNP is the best single predictor of a final diagnosis of HF, compared to individual history, physical examination, chest X-ray, and laboratory findings [44]. Furthermore, the BNP level in the bloodstream has a predictive role for cardiovascular risk in the general population and BNP itself could serve as a therapeutic target for cardiovascular diseases, including hypertension, heart failure, and myocardial infarction [45].

Guidelines recommend the use of BNP or NT-proBNP in the diagnostic algorithm for HF, especially for the patients whose echocardiography was not found to have an important cardiac abnormality [46], with higher levels indicating a higher likelihood for AHF to be the main cause of acute dyspnea [47]. These tests help doctors rule out heart failure quickly and identify those who would benefit from additional confirmatory tests, typically echocardiography, or making echocardiography unnecessary [3, 48]. As the 2016 European Society of Cardiology guidelines for HF recommend, the upper limit of normal in the nonacute setting for BNP is 35 pg/mL and for NT-proBNP it is 125 pg/mL; in the acute setting, higher values should be used (BNP, 100 pg/mL, NT-proBNP, 300 pg/mL, and mid-regional pro-A-type natriuretic peptide (MR-proANP), 120 pmol/L) [46]. However, a cut-point of BNP ≤ 54 pg/mL is recommended for ruling out CHF in severely obese patients (BMI ≥ 35) [49, 50], which indicates that the cut-off points varied among different populations, including the elderly, obese patients, patients with renal disease, and even nonacute patients. According to the 2016 European Society of Cardiology guidelines for HF, when a patient comes into the ED with nonspecific symptoms and signs, such as breathlessness, ankle swelling, and fatigue, there are two alternative paths for clinicians, either echocardiography first or BNP/NT-proBNP first. Because both BNP/NT-proBNP and echocardiography have limitations and the guidelines recommend using them in different situations, it was still worthy of scientific research to explore the association between them Roberts et al. concluded that there was no statistical difference between the diagnostic accuracy of plasma BNP and NT-proBNP, though NT-proBNP had a longer half-life than BNP, and that the measurement of MRproANP may also be a valuable rule-out test for HF [44, 51], which is rarely measured in the current clinical practice. The reason may be that the measurement of ANP in plasma is hampered by marked instability of the hormones, reminding us that biochemical research into detecting the proANP-derived peptides is still worthy of attention [42].

In addition to their utility in HF diagnosis, the levels of BNP or NT-proBNP are remarkably useful for risk stratification and management of patients with suspected HF. In HF management, the trend of decreasing levels of natriuretic peptide indicates effective management strategies [52]. Recently, Troughton et al. reported that for patients aged <75 years with chronic heart failure (CHF), with or without impaired left ventricular systolic function, NP-guided treatment can decrease the all-cause mortality and readmission rate compared with clinically guided therapy, even with its potential to unexpectedly induce or exacerbate concomitant disorders [53]. Across the wide range of HF, even at-risk patients, concentrations of BNP and NT-proBNP also have prognostic value [44]. NT-proBNP can improve the prediction of heart failure in patients with type 2 diabetes [54]. In Piercarlo Ballo's study, the author measured 1012 asymptomatic subjects with systemic hypertension and/or type 2 diabetes with no clinical evidence of HF and concluded that NT-proBNP measurement could provide more information for the prediction of clinical outcome in asymptomatic, stage A-B HF hypertensive and diabetic patients [55]. Furthermore, several studies have established that BNP or NT-proBNP also had strong associations with adverse cardiovascular outcomes in a variety of primary prevention and general populations [37]. In addition, assessment of risk stratification is particularly important in planning end-of-life care for patients and when making the decision to undergo surgery (including transplantation) [3]. Previous meta-analyses have suggested that a single elevated preoperative NP measurement was highly predictive of serious cardiovascular complications after noncardiac surgery [56], and a recent study has indicated that the addition of a postoperative NP measurement significantly improved the prediction of the mortality or nonfatal MI within 30 days or ≥180 days after noncardiac surgery [57]. All these potential functions for the detection of BNP/NT-proBNP levels still need large randomized multicenter trials to better explore this personalized approach to care.

### 2.3. Combined Use of cTn and BNP/NT-proBNP

Both cTn and BNP are quantitative markers of cardiac damage and widely applied in clinical diagnosis, risk stratification, and management of patients with or without cardiovascular diseases. BNP, widely used for the diagnosis and risk stratification of patients presenting with suspected heart failure, may sometimes be measured after an ACS in order to identify patients at high risk and low risk of adverse outcomes [4]. In patients with NSTE-ACS, NT-proBNP has good and comparable predictive value for 30-day mortality [58].

In James and his coworkers' research, they measured BNP in samples obtained three days after the onset of ischemic symptoms in 2525 patients and found that BNP could provide predictive information for use in risk stratification across the spectrum of acute coronary syndromes, such as the risk of new or recurrent myocardial infarction [24]. CTn, commonly used for the diagnosis and risk stratification in patients presenting with suspected ACS, is sometimes measured to help in determining the etiology or trigger of acute HF (AHF). However, there is considerable overlap in cTn levels from AHF without AMI versus AHF with AMI [47]. Age, renal function, diabetes, hypertension, and a history of heart failure have all been reported as determinants of circulating cTnT concentrations [19, 59, 60]. One report has suggested that the levels of NT-proBNP and hs-cTnT may be useful adjuncts to clinical assessment and that both provide much more prognostic information than the total cholesterol or high-sensitivity C-reactive protein levels in this cohort of patients with type 2 diabetes [61]. The prognostic accuracy of the Global Registry of Acute Coronary Events (GRACE) score was improved when combined with three individual biomarkers including hsTnT, NT-proBNP, and high-sensitivity C-reactive protein (hs-CRP) in patients with acute coronary syndrome [62]. However, another study suggested that a dual-biomarker strategy combining the detection of cTn and BNP does not promote the diagnostic accuracy of inducible cardiac ischemia [63].

## 3. Other Clinical Biomarkers

Both cTn and BNP/NT-proBNP are definitive biomarkers of ACS, but they are not enough. Therefore, it is necessary to explore more markers to facilitate earlier and appropriate diagnosis, risk stratification, and management of patients. Moreover, using one or two biomarkers to define the complex pathophysiology of heart failure is inadequate and novel biomarkers are still required for the diagnosis and management of cardiovascular diseases. However, because the literature on the variety of cardiac biomarkers in clinical practice is too large to summarize in one article, this article only focuses on some useful and routine biomarkers.

### 3.1. C-Reactive Protein (CRP)

CRP is a useful prognostic indicator in patients with ACS, as elevated CRP levels are independent predictors of cardiac death, AMI, and congestive heart failure [4]. It is the most widely used inflammatory marker in routine clinical practice. Nonetheless, CRP is a less specific and sensitive biomarker of cardiac injury compared to hs-cTn. Reynoso-Villalpando et al. found that the CRP level was significantly increased in ACS patients compared to the patients without a personal history of ischemic cardiomyopathy [64]. Not only outpatient but inpatient departments often use hs-CRP to assess the outcome in patients with heart disease, such as in MI and ACS [65].

### 3.2. Copeptin

Copeptin, the c-terminal part of the vasopressin prohormone that is released together with arginine vasopressin (AVP) within 0–4 h after symptom onset [66], observably improves baseline cTn sensitivity and could improve effectiveness and safety in combination with cTnT or cTnI for early rule-out of AMI [67–71] without serial testing in comparison to cTn alone [72]. Besides its function of early diagnosis, copeptin was recognized as an independent predictor for all causes of mortality in heart failure patients with reduced ejection fraction, by Pozsonyi and his coworkers [73]. However, as a nonspecific prognostic marker, other conditions could also influence the level of copeptin, such as renal disease and lower respiratory tract infections [69]. Despite this, the 2015 European Society of Cardiology guidelines for NSTE-AC recommended copeptin as a routine clinical examination to clinicians [20], which required more studies to evaluate ideal cut-off levels [69] and the precise roles. However, the high-sensitivity copeptin assay does not seem to increase the diagnostic or prognostic yield already provided by the hs-cTnT assay in the patients suspected of having myocardial infarction in the ED [74].

## 4. The Emerging Biomarkers under Study

### 4.1. Inflammation Biomarkers

#### 4.1.1. Interleukin-6 (IL-6)

IL-6, as well as CRP, is a critical inflammation biomarker that may be implicated in the diagnosis, risk stratification, and prognosis of patients with AMI. IL-6 expression is shown to be elevated in induced myocardial infarction by transcoronary ablation of septal hypertrophy, suggesting its diagnostic role [75]. Additionally, CRP and IL-6 are both significantly upregulated in acute coronary syndrome [76]. The IL-6 concentration, independent of the already established predictors, is also related to adverse cardiac events [77, 78], supporting its potential therapeutic target in unstable ischemic heart disease [78]. The IL-6 receptor antagonist could improve the inflammatory response and the percutaneous coronary intervention- (PCI-) treated cTn release in NSTEMI, and this improvement is independent of the inhibition of the endothelial cell activation [79].

#### 4.1.2. Soluble CD40 Ligand (sCD40L)

sCD40L is a molecule involved in both inflammation and the thrombosis process [80], and a study has shown that it mediates the interaction of platelets and neutrophils [81]. In addition, another study confirms that sCD40L plays a role in the vascular and endothelial dysfunction seen in AMI progression [82]. It has been demonstrated that the sCD40 L concentration is upregulated in AMI patients, accompanied by an increase of the inflammatory biomarker IL-6 and two adhesion molecules, sVCAM-1 and sICAM-1 [83]. However, there are debates over the prognostic performance in patients with ST elevation who underwent PCI. While one study showed that patients with lower sCD40L concentration present with adverse outcomes [84], another study indicates that a high sCD40L level is the cause of increased in-hospital and one-year all-cause mortality rates [85].

#### 4.1.3. Galectin-3 (Gal-3)

Gal-3 is a member of the inflammation mediators and is related to the extent of myocardial inflammation and fibrosis, which is also negatively correlated with ventricular ejection fraction [86]. Furthermore, Gal-3 is thought to be involved in the formation, destabilization, and rupture of plaques [87]. Serum Gal-3 is related to left ventricular dilation and is a contributory factor in predicting the outcome and guiding the monitoring of patients with both acute heart failure and chronic heart failure [88–90].

#### 4.1.4. Other Inflammatory Markers

The interleukin-37 (IL-37) concentration is highly upregulated in acute coronary syndrome and elevated IL-37 baseline is related to poor outcomes [91, 92]. Lipoprotein-associated phospholipase A2 (Lp-PLA2) is another inflammation biomarker that is unsuitable for use as a therapeutic target while its plasma concentration is linked to the risk prediction of cardiovascular outcomes in patients with stable coronary heart diseases [93].

### 4.2. Cardiomyocytes Injury Biomarkers

#### 4.2.1. Cardiac Myosin-Binding Protein C (cMyC)

cMyC is one of the three isoforms of myosin-binding protein C expressed in the cardiac tissue whereas the other two isoforms are expressed in the skeletal muscle [94]. Following cardiomyocyte necrosis, cMyC appears in the circulation earlier compared to hs-cTn [95]. Notably, the cMyC has a higher efficacy for ruling out (safe) and ruling in patients than hs-cTn, while the diagnostic accuracy is similar [96]. Another study also demonstrates that cMyC has discriminatory power comparable to hs-cTn and may perform favorably early after symptom onset [97].

#### 4.2.2. Heart-Type Fatty Acid Binding Protein (hFABP)

hFABP is released from injured myocardium and may serve as a potential biomarker for AMI. The assessment of HFABP at the ED admission adds an incremental value to the initial hs-cTnT. The increase in the sensitivity and negative predictive value for patients with chest pain with noncontributive electrocardiogram could potentially allow safe and early rule-out of AMI without the need for further serial troponin testing [98]. In the patients presenting to the ED with chest pain and no cTnI elevations, hFABP showed a higher sensitivity in the diagnosis of AMI with a positive rate of 55%. It is suggested that hFABP may be a good candidate for AMI rule-in/rule-out within the ED context [99]. The addition of hFABP to hs-cTn has more accuracy and accelerates clinical diagnostic decisions by identifying the patients at low risk for AMI [100, 101]. Another study has demonstrated that hFABP has superior sensitivity than cTnI and suggests a combination use of hFABP and cTnI subsequently to diagnose AMI [102].

#### 4.2.3. Endothelial Cell Related Biomarkers

Endothelial cell-specific molecule 1 (ESM-1), also known as endocan, is a biomarker for endothelial dysfunction [103] and may serve as a novel evaluation method for risk stratification of patients with acute STEMI [104]. Another study also demonstrated that high endocan level at presentation was an independent predictor for adverse cardiac events [105, 106].

#### 4.2.4. Platelet Related Biomarkers

Mean platelet volume (MPV) and beta-thromboglobulin (beta-TG) are two important platelet biomarkers that may increase during platelet activation and have a higher expression in patients with coronary artery diseases [76]. High MPV value also correlates with an increased incidence of long-term adverse events, especially all-cause mortality in NSTEMI patients undergoing PCI [107]. Additionally, research demonstrates that MPV to platelet count ratio (MPV/P ratio) has a good prognostic performance in predicting the outcomes of patients with AMI [108, 109], which is similar to the GRACE score, with the MPV/P ratio being easier to calculate [110]. The MPV/P ratio also has superior performance compared to MPV alone in predicting adverse outcomes in patients with NSTEMI undergoing PCI primarily [110, 111]. Platelet microRNA-126 may also work as a novel biomarker for AMI; however, the low correlation between its expression and platelet activity limits its diagnostic utility [112].

### 4.3. Other Biomarkers

#### 4.3.1. Suppression of Tumorigenicity 2 (ST2)

ST2 is a member of IL-1 receptor family with two forms, namely, transmembrane (ST2L) and soluble (sST2) isoforms [113]. sST2 has been shown to have high expression in patients with acute myocardial infarction, accompanied by the elevation of GDF-15, HFABP, and suPAR, and downregulation of Fetuin A [114]. Elevated ST2 concentration is a predictor of major adverse cardiac events in ACS patients [115]. The serum ST2 level positively correlates with IL-33 and BNP levels, and all of these biomarkers are independent predictors of major adverse cardiac events in AMI patients undergoing PCI [116]. The 2017 ACCF/AHA guidelines for the Management of Heart Failure have specified ST2 receptors and galectin-3 as prognostic biomarkers for the prediction of hospitalization and death and to provide additional prognostic value in patients with HF [117]. Animal model research has confirmed that IL-33 prevents cardiomyocyte apoptosis and thereby improves cardiac function after MI [118]. Additionally, modulation of the IL-33/ST2 signal may also exert a beneficial effect against the adverse postinfarction cardiac remodeling [119–121].

In addition to MI, sST2 also has a beneficial role in diagnosing heart failure, especially for patients with high IL-33 levels [122]. Also, the sST2-assist score enables the prediction of left ventricular reverse remodeling in systolic heart failure patients [123, 124], and the sST2 concentration is highly related to cardiac death and readmission for worsening heart failure [125, 126]. However, it should be noted that when adjusted for NT-proBNP, the prognostic information of baseline sST2 diminishes [127, 128]. The measurement of sST2 also contributes to the risk stratification for long-term HF and is superior to Gal-3, whose contribution to clinical risk factors is trivial [129].

#### 4.3.2. Cystatin C (cys-C)

cys-C is generated in almost all human nucleated cells and serves as a biomarker for early renal impairment. An elevated cys-C level is related to impaired coronary perfusion and undesirable recovery of cardiac function in STEMI patients undergoing PCI [130] and predicting adverse outcomes [131–133]. A meta-analysis also demonstrated that increased cys-C concentration is positively correlated with readmission rates and all-cause mortality in HF patients [134].

#### 4.3.3. miRNAs

miRNA-208 and miRNA-499 are solely expressed in cardiomyocytes. AMI patients exhibit significant increases in the concentrations of circulating miRNA-208b and miRNA-499 compared with the health control group [135–137]. In terms of diagnosis, there is a correlation between microRNA-208b and plasma cTnT concentration. However, miR-208b provides lower diagnostic accuracy than miR-499 and hs-cTnT. miR-208b and miR-499 are inversely proportional to ejection fraction and can be used as a prognostic biomarker for left ventricular dysfunction after MI [135].

miRNA-1 and miRNA-133a are muscle-specific microRNAs that regulate cardiomyocyte growth and differentiation [138]. They are abundantly expressed in both skeletal and cardiac muscles and human cardiomyocytes and have been shown to be involved in the regulation of cardiac hypertrophy [139]. Serum miRNA-1 and mi-RNA133a levels were significantly elevated in a group of patients with unselected AMI. Circulating miRNA-133a can be used as a marker for cardiomyocyte death, and it may have functions in cardiovascular diseases [140].

#### 4.3.4. Long Noncoding RNAs (lncRNAs)

lncRNAs, as well as miRNAs, are observed to be altered in patients with myocardial infarction and may be beneficial for the diagnosis of myocardial infarction [141–143]. In addition, the plasma levels of lncRNAs are confirmed to provide prognostic information for myocardial infarction and predict future death in patients with heart failure [144, 145].

#### 4.3.5. Sirtuin (SIRT)

The SIRT family, comprising seven proteins (SIRT1–SIRT7), attracted attention as stress adaptors and epigenetic enzymes involved in the cellular events controlling aging-related disorders and cardiovascular disease [146]. Among sirtuins, SIRT1 is the best characterized protein for its protective roles against inflammation, vascular aging, heart disease, and atherosclerotic plaque development. A whole-genome expression analysis revealed that SIRT1 transcription levels were reduced in peripheral blood monocytes isolated from patients affected by acute myocardial infarction, unstable angina, and overall ACS. The authors also showed that SIRT1 mRNA expression was negatively associated with the gene expression of interleukine-6 [147]. SIRT1 mRNA expression was significantly downregulated in the failing heart [148]. Endogenous SIRT1 plays a pivotal role in mediating the cell death/survival process and has been implicated in the pathogenesis of cardiovascular diseases.

#### 4.3.6. Triggering Receptor Expressed on Myeloid Cells (TREML)

TREMLs are important effectors of the innate immune system, and polymorphisms within genes encoding them may increase the risk of occurrence of various pathologies including cardiovascular disorders. The analysis of the whole genomic expression in the peripheral blood cell model showed that TREML4 was upregulated in the early stage of the acute coronary syndrome [149], which indicated that TREML4 might be used as a biomarker in the early stage of ACS and monitor the recovery of early myocardial ischemia [150]. TREML1 expression is upregulated in ischemic myocardium. The activated form of TREML1 can be detected in the plasma of patients with acute myocardial infarction, the concentration of which is an independent predictor of death. TREML1 genetic or pharmacological inhibition dampens myocardial inflammation and improves left ventricular function and survival [151].

#### 4.3.7. Growth-Differentiation Factor-15 (GDF-15)

GDF-15 is a member of the transforming growth factor-*β* (TGF-*β*) cytokine superfamily that is widely expressed and may be induced in response to tissue injury. Under pathological conditions, GDF-15 can be produced by many cardiovascular and noncardiovascular cell types. Higher circulating GDF-15 levels are associated with increasing cardiometabolic risk factors in individuals without the overt cardiovascular disease [152]. GDF-15 expression is dramatically upregulated in cardiovascular disease, and the incidence of cardiovascular events is positively correlated with the concentration of GDF-15 [153, 154], suggesting its potential value as a disease biomarker. Meta-analysis shows that high levels of GDF-15 may increase the risk of mortality in patients with cardiovascular diseases [155, 156]. GDF-15 is an independent predictor of all-cause mortality in ACS patients, adding incremental value to traditional risk factors and to NT-proBNP and C-reactive protein levels [157, 158].

#### 4.3.8. Pregnancy-Associated Plasma Protein-A (PAPP-A)

PAPP-A is a high molecular weight and zinc-binding metalloproteinase, and several studies have demonstrated that PAPP-A plays a role in cardiovascular diseases. PAPP-A is a sensitive, specific, and early marker for ACS diagnosis. Coronary PAPP-A levels were significantly elevated among patients at risk for cardiovascular diseases [159]. In the early stages of STEMI, the sensitivity of PAPP-A was superior to that of CK-MB and troponin T [160]. Even in cTnI-negative patients with the acute coronary syndrome, elevated PAPP-A can be used as an independent predictor of adverse outcomes [161]. Meta-analysis showed that PAPP-A is also an independent risk factor for all-cause mortality or cardiovascular events. Moreover, this positive correlation was not affected by the different follow-up periods, coronary artery disease types, and PAPP-A detection methods [162].

#### 4.3.9. Others

The class II phosphatidylinositol 3-phosphate kinase (PIK3C2A) and the protein arginine methyltransferase 5 (PRMT5) expression have been verified to be downregulated in the AMI and are independent risk factors [163, 164]. YKL-40 is a novel inflammatory biomarker in cardiovascular disease [165], and the high plasma levels of cys-C are associated with the spectrum of adverse outcomes and risk stratification in cardiovascular diseases [166]. There are many new biomarkers with superior sensitivity and specificity for scientists to explore, and for the known biomarkers, there is still a challenge for the scientific community to apply the information towards patient monitoring, diagnosis, or replacement of current biomarkers for cardiovascular diseases. We summarize the characteristics and clinical uses of each biomarker in Table 1.

## 5. Conclusions

Currently, clinicians typically measure either cTn or BNP/NT-proBNP when encountering patients with suspected acute coronary syndromes or heart failure, based on the guidelines published by the European Society of Cardiology or American Heart Association [46]. Knowing patients with other system diseases, such as renal diseases and diabetes, who are at considerable risk of vascular complications especially asymptomatic cardiovascular disease, the levels of cTn or BNP/NT-proBNP should be measured regularly to assess the risk stratification and take the preventive steps in time. Perhaps the cTnT or cTnI should be measured separately from the total cTn complex, or cTn might be combined with BNP/NT-proBNP to take advantage of their surprising roles in early diagnosis or long-term prognosis in patients with ACS. The different roles and applications of cTn, BNP/NT-proBNP and others in different situations should be confirmed via various experimental research and clinical analyses. All in all, there is robust evidence to support the role of troponin and BNP as biomarkers in clinical medicine, but there could be some limitations. Thus, complementary biomarkers could be useful. We still need to identify more novel biomarkers to supplement ECG or X-rays and provide more accurate and sensitive methods for cardiovascular disease diagnosis, risk stratification, and management.

## Figures and Tables

**Table 1:** Clinical application of biomarkers.

Biomarker	Pathophysiology	Clinical usefulness
hs-cTn	Myocardial injury	Diagnostic
		Prognostic
		Risk stratification
		Therapeutic guidance

BNP/NT-proBNP	Myocyte stretch	Diagnostic
		Prognostic
		Risk stratification
		Therapeutic target
		Therapeutic guidance

CRP	Inflammation	Prognostic

Copeptin	Oxidative stress	Diagnostic
		Prognostic

IL-6	Inflammation	Diagnosis
		Prognostic
		Risk stratification

sCD40L	Inflammation	Potential prognostic value

Gal-3	Hypertrophy/fibrosis	Prognostic
		Therapeutic guidance

cMyC	Myocardial injury	Diagnostic

hFABP	Myocardial injury	Diagnostic

ESM-1	Endothelial dysfunction	Risk stratification
		Prognostic

MPV	—	Prognostic

ST-2	Hypertrophy/fibrosis	Risk stratification
		Prognostic
		Therapeutic guidance

cys-C	Myocardial injury	Prognostic

miRNA	—	Potential diagnostic and prognostic value

lncRNA	—	Potential prognostic value

SIRT	Oxidative stress	Potential prognostic value

TREM	Inflammation	Potential prognostic value

GDF-15	Apoptosis	Potential prognostic value

PAPP-A	Myocardial injury	Diagnostic
		Prognostic

BNP: B-type natriuretic peptide; cMyC: cardiac myosin-binding protein C; CRP: C-reactive protein; cys-C: cystatin C; ESM-1: endothelial cell-specific molecule 1; Gal-3: galectin-3; GDF-15: growth-differentiation factor-15; hFABP: heart-type fatty acid binding protein; hs-cTn: high-sensitivity troponin; IL-6: interleukin-6; lncRNA: long noncoding RNA; miRNA: microRNA; MPV: mean platelet volume; NT-proBNP: N-terminal probrain natriuretic peptide; PAPP-A: pregnancy-associated plasma protein-A; sCD40L: soluble CD40 ligand; SIRT: sirtuin; ST-2: suppression of tumorigenicity 2; TREML: triggering receptor expressed on myeloid cells.

## Supplementary Materials

Supplementary Materials Table S1 contains a number of clinical trials evaluating the role of cardiac troponin in acute coronary heart disease. Table S2 summarizes the current clinical trials cited in the B-type natriuretic peptide topic. Table S3 summarizes the diagnostic and prognostic value of C-reactive protein and copeptin in patients with acute myocardial infarction. Recently, some emerging biomarkers of acute myocardial infarction and heart failure have received increasing attention, and we have summarized them in Table S4.
