# Supplementary material for: The Biomarkers for Acute Myocardial Infarction and Heart Failure
Source: Biomed Res Int. 2020 Jan 16;2020:2018035. doi: 10.1155/2020/2018035 (PMC6988690; doi:10.1155/2020/2018035)
Supplement: Supplementary Materials — Table S1 contains a number of clinical trials evaluating the role of cardiac troponin in acute coronary heart disease. Table S2 summarizes the current clinical trials cited in the B-type natriuretic peptide topic. Table S3 summarizes the diagnostic and prognostic value of C-reactive protein and copeptin in patients with acute myocardial infarction. Recently, some emerging biomarkers of acute myocardial infarction and heart failure have received increasing attention, and we have summarized them in Table S4. [file 2018035.f1.pdf]

Table S1: Summary of clinical trials for cardiac troponin

First author	Year	Biomarker (s)	Study design	Findings	Ref.
Shah AS	2015	hs-cTnI	prospective	In 30 days,the NPV of troponin concentration <5 ng / L for MI or cardiogenic death is 99.6%;At 1 year, risk of MI and cardiac death was lower in patients with cTn<5 ng/L than cTn >5 ng/l(0.6% vs 3.3%,p < 0.001)	21
Grinstein J	2015	hsTnT	prospective	hsTnT ≥14 ng/L carried a 5.2-fold higher risk of CVD or MI ;The hsTnT concentration of 14-50 ng / L was more than 3.3-fold higher short-term risk of CVD or recurrent MI compared to patient at <14 ng/L	22
Magnoni M	2018	hsTnT	prospective	At 30 and 180 days, patients with hsTnT ≥14 ng/L had a higher rate of CVD or MI (30-day: 5.9% vs 0.8% p = 0.001; 180-day: 11.1% vs 4.7% p = 0.002).	23
Haaf P	2014	hs-cTnT<br>hs-cTnI	prospective	hs-cTnT predicts mortality more accurately than hs-cTnI assays in patients with suspected AMI.（0.78vs0.71);<br>Changes of hs-cTn did not further improve risk stratification beyond initial presentation values	27
Boeddinghaus J	2017	hs-cTnT<br>hs-cTnI	prospective	In patients with mild hs-cTnT/I(hs-cTnT:26.2 ng/L-75ng/L;hs-cTnI:14 ng/L-50ng/L),diagnostic accuracy for AMI is 0.51 for hs-cTnI,0.78 for 1h-hs-cTnI changes	28
Pickering JW	2016	hs-cTnT<br>hs-cTnI	prospective	The ESC-rule-in algorithm has good specificity and can rule-in approximately 60% of AMIs.<br>The sensitivity to rule-out AMI is too low for clinical use	29
Boeddinghaus J	2018	hs-cTnT<br>hs-cTnI	prospective	Rule-out safety(young 100%, middle 99.3%, old 99.3%) of the ESC hs-cTnT 0/1h-algorithm was very high in all age-strata;Accuracy of rule-in(young 97%, middle 96.1%, old 92.7%) and triage efficacy(young 93%, middle 80%, old 55%) decreased with age	30
Mueller-Hennessen M	2016	hs-cTn	prospective	Age-specified cut-offs reclassified patients for outcomes of 1-month and 3-month mortality in the ACS cohort (p<0.001)<br>No significant differences in outcomes could be found using gender-specific cut-offs.	31
Yang S	2016	hs-cTnT	prospective	According to the age and gender tailored cutoff value, the specificity and positive predictive value of AMI diagnosis were increased from 53.9% to 72.2% and 48.6% to 60.8%	33
Rubini Gimenez M	2014	hs-cTnT/I	prospective	Compared with hs-cTnT, early reporters (<3 h) hs-cTnI showed higher diagnostic accuracy (0.92 vs 0.89, P = 0.019), while in late presenters hs-cTnI was superior than hs-cTnI (0.96 vs 0.94, P = 0.007); hs-cTnT had a higher prognostic accuracy for all-cause mortality compared with hs-cTnI. (0.8vs 0.75, P <0.001)	34
Klinkenberg LJ	2016	hs-cTnT/I	prospective	hs-cTnT exhibited a diurnal rhythm, the diurnal hs-cTnT rhythm does not affect the diagnostic accuracy of hs-cTnT for AMI (all AUC >0.93);hs-cTnI exhibited no diurnal rhythm with no differences in AUC among early-morning and evening presenters.	35
Wildi K	2018	hs-cTnI	prospective	Diagnostic accuracy for AMI of hs-cTnI assays were high and comparable between patients presenting in the morning versus presenting in the evening	36
Everett BM	2015	hs-cTnI<br>BNP	prospective	hsTnI concentrations（M≥4.6ng/L,F≥3.9ng/L) and BNP levels （M≥28.6ng/L,F≥44.4ng/L) were associated with a first major cardiovascular event;The risk of all-cause mortality was elevated for the highest versus the lowest tertiles of hsTnI and BNP .	38
Árnadóttir Á	2018	hs-cTnT/I	prospective	The relationship between elevated hs-cTn and mortality was strong for both hs-cTnT and hs-cTnI (HR 6.0 vs. 5.1).<br>The prognostic accuracy for long-term mortality was superior for hs-cTnT than for hs-cTnI (AUC 0.81 vs 0.74, p < 0.001).	39

ACS: acute coronary syndrome; AMI: acute myocardial infarction; AUC: area under the curve; BNP: B-type natriuretic peptide; CVD: cardiovascular death; ESC: European Society of Cardiology; F:female; hs-cTn: high sensitivity troponin; HR: hazard ratios; M:male; MI: myocardial infarction; NPV: negative predictive value

Table S2: Summary of clinical trials for BNP/ NT-proBNP and combined use of cTn and BNP/NT-proBNP.

First author	Year	Biomarker (s)	Study design	Findings	Ref.
Ohkuma T	2017	IL-6<br>hs-CRP<br>hs-cTnT<br>NT-proBNP	nested case-cohort study	Addition of NT-proBNP to a model including conventional risk factors improved discrimination and classification of the 5-year risk of HF(0.8162 to 0.8800, P <0.001);NT-proBNP alone showed comparable predictive ability compared with conventional risk factors (0.8239 vs. 0.8162, P = 0.74).	55
Ballo P	2016	NT-proBNP	prospective	NT-proBNP adds independent and incremental prognostic information to a predictive model (p < 0.0001).<br>This prognostic value is further evident in the elderly and among women.	56
Schellings DA	2016	NT-proBNP<br>hs-cTn	prospective	The predictive value of NT-proBNP did not differ significantly compared to the GRACE risk score (AUC: 0.85 vs 0.87, p=0.67);Adjustment of the GRACE risk score by adding NT-proBNP did not improve prognostication	59
Hillis GS	2014	NT-proBNP<br>hs-cTn	nested case-cohort study	The addition of NT-proBNP or cTnT improved 5-year risk classification for cardiovascular events (39% for NT-proBNP and 46% for hs-cTnT);The combination of NT-proBNP and cTnT provided optimal risk discrimination.	62
Klingenberg R	2018	hsTnT<br>NT-proBNP<br>hsCRP<br>PIGF<br>sFlt-1	prospective	The prognostic accuracy of the GRACE score was improved when combined with hsTnT, NT-proBNP and hsCRP to yield a 9% increment (C-statistic 0.73->0.82) for the discrimination of short-term risk for all-cause mortality.	63
Puelacher C	2018	NT-proBNP<br>hs-cTn	prospective	The combination of hs-cTnI and BNP with CJb did not provide a significant advantage over the combination of hs-cTnI alone and CJb (AUC 0.74 vs AUC 0.74, p = 0.16).<br>Hs-cTnI showed good prognostic value for AMI (HR 1.6, 95%CI 1.3–1.9), and BNP for death (HR 1.6, 95%CI 1.3–2.1).	64

AUC: area under the curve; CJb: clinical judgment before exercise stress testing; GRACE: Global Registry of Acute Coronary Events; HF: heart failure; HR: hazard ratios; hs-CRP: high sensitivity C-reactive protein; hs-cTnT: high sensitivity troponin T; IL-6: interleukin-6; NT-proBNP: N-terminal pro-brain natriuretic peptide; PIGF: placental growth factor; sFlt-1: soluble fms-like tyrosine kinase-1

Table S3: Summary of clinical trials for other clinical biomarkers

First author	Year	Biomarker (s)	Study design	Findings	Ref.
Reynoso-Villalpando G	2017	CRP<br>IL-6	unknown	Serum CRP was increased in ACS patients(p<0.0001).<br>STEMI exhibited a higher CRP concentration than NSTEMI and patients with UA (21.81,17.10,and 5.91 mg/L; p < 0.01).	65
Afzali D	2013	copeptin<br>TnI	prospective	Copeptin level was higher in AMI patients(p<0.0001).<br>A troponin I level <0.04 ng/mL in combination with copeptin <14 pmol/L at admission ruled out AMI with an NPV of 97.3 %;<br>Copeptin as strong predictor of intermediate-term mortality (HR 4.28,p = 0.004).	67
Folli C	2013	copeptin<br>TnI	prospective	The combination of copeptin and troponin-T attained a NPV of 86.6% for ACS, of 97.9% for other potentially life-threatening non-ACS diseases and of 85% for all potentially lethal diseases (ACS plus others).	69
Jeong JH	2020	CK-MB<br>TnI<br>copeptin	prospective	Copeptin had better diagnostic performance than TnI in patients with chest pain within one hour of onset (AMI: P=0.022, ≤1 hour; STEMI: P=0.017, ≤1 hour);TnI and copeptin in combination exhibited better diagnostic performance than CK-MB plus TnI in AMI and STEMI patients.	71
Abd El Baky Mahmoud M	2018	CK-MB<br>cTnI<br>copeptin	unknown	The accuracy of copeptin in the early diagnosis of AMI is higher than that of CKMB and troponin(Z = 5.29, P < 0.001).<br>ROC curve analysis of serum Copeptin for discriminating AMI group from UA group showed diagnostic sensitivity and specificity of 100%.	72
Pozsonyi Z	2015	copeptin	prospective	Copeptin is an independent long-term prognostic marker in HFREF(HR 2.168);The addition of copeptin to the predictive model resulted in a minor (8.21%) improvement, whereas the final, multivariable model showed a significant increase in net reclassification (10.26%, p=0.015).	74
Alquézar A	2017	hs-copep<br>hs-cTnT	retrospective	The ROC of hs-cTnt combined with hscope assay was not better than the ROC for the hs-cTnt by itself (P=0.89).<br>Elevated hs-copep findings did not provide prognostic information that was not already provided by hs-cTnt findings (P=0.56).	75

CK-MB: creatine kinase isoenzyme MB; CRP: C-reactive protein; HFREF: heart failure with reduced ejection fraction; HR: hazard ratios; hs-cTnT: high sensitivity troponin T; IL-6: interleukin-6; NPV: negative predictive value; NSTEMI: non-ST-segment elevation myocardial infarction; STEMI: ST-segment elevation myocardial infarction; TnI: troponin I; ROC: receiver operating characteristic curve; UA: unstable angina

Table S4: Summary of studies for the emerging biomarkers under study

First author	Year	Biomarker (s)	Study design	Findings	Ref.
Liebetrau C	2015	CRP<br>hs-CRP<br>IL-6<br>Scd40l	unknown	Serum levels of CRP were increased at 24 hours, whereas hs CRP increased as early as 8 hours.IL-6 was increased at 45 minutes, and sCD40l was decreased at 60 minutes.	76
Kamińska J	2018	IL-6,β-TG<br>PLT, MPV<br>LPLT, WBC	unknown	WBC, CRP, IL-6, MPV and β-TG in ACS patients were higher than those of healthy people.	77
Held C	2017	IL-6<br>hs-CRP	prospective	L-6 was associated with increased risk of MACE (HR:1.60, P<0.0001); cardiovascular death (HR, 2.15, P<0.0001);MI (HR, 1.53,P<0.05); all-cause mortality (HR, 2.11,P<0.0001); and risk of hospitalization for HF (HR, 2.28,P<0.001).	78
Fanola CL	2017	IL-6	retrospective	For every SD increase in IL-6, there was a 10% higher risk of MACE and a 22% higher risk of cardiovascular death or HF.<br>IL-6 (>3.97 pg/mL) had a higher risk of MACE and cardiovascular death or HF.	79
Kleveland O	2016	hs CRP<br>hsTnT	double-blind, placebo-controlled trial	The median AUC for hs-CRP during hospitalization was 2.1 times higher in the placebo than in the tocilizumab group (4.2 vs. 2.0 mg/L/h, P < 0.001).<br>The median AUC for hsTnT during hospitalization was 1.5 times higher in the placebo group compared with the tocilizumab group (234 vs. 159 ng/L/h, P = 0.007).	80
Setianto BY	2010	blood count<br>sCD40L	unknown	Neutrophil count was higher in AMI as compared with UA (P < 0.001), whereas sCD40L did not significantly differ;There is a strong and positive significant correlation between neutrophil count and sCD40L level (r = 0.607, P = 0.002) in UA.	82
Napoleão P	2015	sCD40L<br>eNOS<br>VEGF<br>CD62P	unknown	Time changes of sCD40L over 1 month after MI onset were associated with G894T eNOS polymorphism and with the VEGF concentrations, but not to the platelet CD62P expression.	83
Tousoulis D	2007	sCD40L<br>IL-6<br>sVCAM-1<br>sICAM-1	prospective	Both CAD and AMI are accompanied by increased levels of sCD40L(P < 0.01).Parallel with an elevation of proinflammatory cytokine IL-6(P < 0.01) and adhesion molecules sVCAM-1 and sICAM-1. (P < 0.05)	84
Pusuroglu H	2014	sCD40L	prospective	Higher in-hospital and all-cause mortality in patients with sCD40L >0.947 mg/l (7.7 vs. 3.3%, P=0.029; 16.1 vs. 4.8%, P<0.001, respectively).sCD40L value at admission (>0.947 mg/l) is a powerful independent predictor of 1-year all-cause mortality (odds ratio: 3.68;P=0.003).	85
Kang Q	2018	Gal 3	unknown	Gal 3 level of the AMI group was higher than that of the UAP group and SAP group (P<0.05);Gal 3 level of multivessel coronary disease group was higher than that of single vessel group (P<0.05);Galectin-3 was negatively correlated with the LVEF value(r=-0.405, P<0.05).	86
Bivona G	2016	Gal 3	unknown	The concentration of Gal 3 is higher in AMI patients within 1h from admission, and lower at discharge(18 vs 16.8,p = 0.006).<br>Gal 3 levels were correlated to hsTnI and eGFR on admission (r = 0.2; p <0.001 and r = -0.25; p <0.001, respectively).	88
French B	2016	Gal 3	prospective	Gal 3 levels were associated with risk among participants with preserved LVEF (RR:3.30, P<0.001).<br>Gal 3 can accurately discriminator of risk among participants with preserved LVEF in 5 years(AUC:0.782)	89
Zivlas C	2017	CysC<br>Gal 3	unknown	Plasma CysC was positively correlated with LAVi (R2 = 0.135, p = 0.019) and log-transformed plasma Gal-3 (R2 = 0.109, p = 0.042) ;MRV (t = 2.236, p = 0.032), CysC (t = 2.467, p = 0.019) and RVSP (t = 2.155, p = 0.038) were significant predictors of LAVi.	91
Liu K	2017	IL-37	prospective	The circulating concentration of IL-37 was higher in the ACS patients than in either of the normal or SAP patients (p<0.05), IL-37 ⩾ 341.1 pg/ml was independent predictors of in-hospital MACE (p < 0.05).	92
Yang T	2017	IL-37	unknown	The mortality rate was lower in patients with IL-37 serum<6.4 pg/mL than those with IL-37 serum >6.4 pg/mL at 36-month follow-up (16% vs. 24%, p=0.02, log rank X2=5.39).	93

Wallentin L	2016	Lp-PLA2	prospective	Comparing the highest(230μmol/min/L) and lowest(125μmol/min/L) Lp-PLA2 groups, the HR were 1.50 for the primary composite end point (CV death, MI, or stroke). There were no associations between on-treatment Lp-PLA2 activity or changes of Lp-PLA2 activity and outcomes.	94
Kaier TE	2017	cMyC<br>hs-cTnT<br>hs-cTnI<br>cTnI	prospective	The discrimination of cMyC for AMI, as quantified by the AUC, was 0.924, compared to the AUCs for hs-cTnT 0.927, hs-cTnI 0.922 ;cMyC was superior to hs-cTnI and standard sensitivity cTnI (P<0.05 for both) and similar to hs-cTnT at predicting death at 3 years.	98
Dupuy AM	2015	hs-cTnT<br>h-FABP	prospective	Adding h-FABP to hs-cTnT at 5.8-ng/mL and 14-ng/L thresholds, respectively, increased both sensitivity and NPV for NSTEMI diagnosis, with about 13% and 3% increase,leading to a sensitivity of 97% and an NPV of 99%	99
Agnello L	2017	h-FABP<br>hs-Tn	prospective	Among AMI patients, 55% were positive for h-FABP and 34.6% were positive for hs-TnI (p=0.015). h-FABP showed a higher sensitivity(55.5% vs 34%) but lower specificity(89.2% vs 100%) than hs-TnI.	100
Joanna M. Young	2016	h-FABP<br>hs-cTnT<br>hs-cTnI	unknown	H-FABP<4.3 ng/mL plus hs-cTnI<10.0 ng/L together with a negative ECG maintained >99 % sensitivity for AMI whilst classifying 40.9 % of patients as low-risk. The combination of H-FABP<3.9 ng/mL and hs-cTnT<7.6 ng/L with a negative ECG maintained the same sensitivity whilst classifying 32.1 % of patients as low risk	102
Vupputuri A	2015	h-FABP<br>CK-MB<br>cTnI	prospective	The sensitivity and specificity of h-FABP was 89.7% and 68% for diagnosis of AMI. The sensitivity of h-FABP was superior to initial cTnI and CK-MB, for AMI patients within 6h (100% vs 46.1%, 33% respectively).	103
Qiu CR	2017	hsCRP<br>ESM-1	unknown	Serum ESM-1 levels were higher in the AMI group (P<0.05). In patients with AMI, serum ESM-1 levels were not significantly correlated with hsCRP levels. There was no significant correlation between serum ESM-1 level and Gensini score.	104
Kundi H	2017	hsCRP<br>endocan	unknown	Endocan independently correlated with the presence of STEMI. A cutoff endocan level of 1.7 (ng/mL) predicted the presence of STEMI with a sensitivity of 76.1% and specificity of 73.6%.	106
Qiu C	2016	ESM-1	unknown	Serum ESM-1 level was higher in patients with stress hyperglycemia patients having STEMI (P <0.05). serum ESM-1 levels >1.01 ng/mL (odds ratio 3.01, 95% confidence interval 1.05-8.64, P <0.05) were an independent predictor of MACEs.	107
Wasilewski J	2016	MPV	retrospective	The 12-month all-cause mortality was higher along with an increasing level of MPV (6.7% vs. 5.5% vs. 10.0% vs. 12.8% in each subsequent quartile of MPV; p = 0.0047). The level of MPV was an independent predictor of death or non-fatal MI (adjusted HR 1.16; 95% CI 1.03–1.31; p = 0.017).	108
Yu T	2017	MPV/P	retrospective	MPV/P⩾0.054 was an independent predictor of all-cause mortality (HRs: 1.973, P<0.001), and all-cause mortality/nonfatal myocardial reinfarction (HRs: 1.289, P<0.001);The discriminatory performance of MPV/P ratio was similar to GRACE score but better than MPV	111
Tian C	2018	MPV/PC	unknown	MPV/PC⩾ 0.055 was an independent predictor of MACE(HR: 1.121, P < 0.01), all-cause mortality (HR: 1.109,P = 0.020) MPV/PC has good accuracy for predicting MACE (AUC: 0.764) MPV/PC was better than MPV for predicting MACE (MPV/PC ratio versus MPV: z = 2.285, P = 0.022), in patients with STEMI undergoing P-PCI.	112
Li S	2017	pmiRNAs	prospective	Expressions of pmiR-21 and pmiR-126 were decreased, while pmiR-150 and pmiR-223 were increased in STEMI patients when compared to healthy volunteers (all p<0.01). pmiR-126 exhibited correlation with plasma cardiac troponin I (r = - 0.556, p = 0.011) in STEMI.	113
Schernthaner C	2017	sST2<br>GDF-15<br>h-FABP<br>suPAR	retrospective	Plasma levels of novel biomarkers were elevated (sST2, GDF-15, h-FABP, suPAR) or inversely downregulated (fetuin A) in patients with AMI compared to a control group with excluded coronary artery disease.(P<0.001,respectively)	115
Jha D	2018	sST2	prospective	AUC for predicting MACE occurrence in ACS patients was 0.72 (P = 0.04). The optimal cut-off value for sST2 was 36.5 ng/mL with 87.5% sensitivity and 71.7% specificity.	116

Wang YP	2017	ST2<br>IL-33<br>BNP	unknown	Serum levels of ST2, IL-33 and BNP were positively correlated with each other in all AMI patients (r = 0.22, r = 0.42, r = 0.23, all p<0.05);1-year overall survival rate was higher in AMI patients with low serum levels of ST2 (≤ 733.82 pg/ml), IL-33 (≤ 387.75 pg/ml) and BNP (≤ 285.73 pg/ml) than those with high serum levels of ST2, IL-33 and BNP (all p<0.05).	117
Seki K	2009	/	Animal model	IL-33 reduced cardiomyocyte apoptosis, suppressed caspase-3 activity;IL-33 decreased both infarct and fibrosis volumes at 15 days;IL-33 improved ventricular function.;IL-33 improved survival after MI in wild-type but not in ST2(-/-) mice.	119
Chen B	2018	/	Animal model	MI group had higher level of IL-33, sST2, and IL-1β, as well as higher concentrations of markers of fibrosis and inflammation; IL-1β could induce expression of sST2, accelerating the progression of heart failure after acute MI;Eplerenone could improve LV function by reducing expression of IL-1β and sST2.	120
Sánchez-Más J	2014	/	Animal model	The mRNA levels of IL-33 and sST2 were upregulated in the infarcted myocardium during the first week after AMI.<br>IL-33 levels remained elevated during the first 12 weeks post-AMI, sST2 levels showed a marked drop at 4 weeks.<br>The expression of sST2 positively correlated with cardiac gene expression of inflammatory and fibrosis markers.	122
Luo NS	2017	s-ST2<br>IL-33	unknown	Serum concentrations of IL-33 and sST2 were higher in HF-pEF patients than in patients in non-HF group (median, IL-33: 0.437 μg/L vs. 0.127 μg/L, P<0.01; sST: 0.118 μg/L vs. 0.067 μg/L, P<0.01);The AUC of sST2 for detecting HF-pEF was higher than NT-proBNP in population with high serum IL-33 (AUC: 0.88 vs. 0.83, P<0.01).	123
Lupón J	2016	ST2	unknown	ST2-R2 score was related to the changes of LVEF and indexed LV sizes;HR for risk of death, using the lower ST2-R2 score strata (<9) as a reference, were 0.49 (p<0.001; score 9-11), 0.27 (p<0.001; score 12-14), and 0.17 (p<0.001; score 15-17)	124
Bahuleyan CG	2018	sST2	prospective	sST2 concentration was higher among patients with adverse events (p≤0.001).<br>Optimal cut-off value to predict cardiac death and re-hospitalization of sST2 is 49ng/ml, with a sensitivity and specificity of 72% and 75%, respectively.	126
Mueller T	2016	BNP<br>galectin-3<br>sST2	prospective	BNP had a higher AUC for the diagnosis of HF (0.92) than galectin-3 (0.57) and sST2 (0.63).<br>The AUC of BNP for the prediction of one-year all-cause mortality in HF patients (0.72) was not different from the AUCs of galectin-3 (0.70) and sST2 (0.75).	127
Tang WH	2016	sST2<br>NT-proBNP	prospective	Higher sST2 levels were associated with increased death risk at 180 days (baseline HR: 2.21; follow-up HR: 2.64; both p < 0.001); Prognostic value of baseline sST2 diminished after adjusting for clinical covariates and aminoterminal pro-BNP	129
Bayes-Genis A	2014	ST2<br>Gal-3	unknown	ST2 is independently associated with cardiovascular mortality (hazard ratio: 1.27, p = 0.014).<br>Incorporation of ST2 into a full-adjusted model for all-cause mortality improved discrimination (C-statistic: 0.77, p = 0.004) and calibration, and reclassified significantly better	130
Tang L	2016	Cys-C	prospective	The incidence of CHF in patients with Cys-C≥ 1.36 mg/L was higher than Cys-C < 1.36 mg/L. (18.5 vs. 5.6 %, p = 0.022).<br>Cys-C levels at admission were a independent predictor of angiographic no-reflow and the development of CHF at 6-month follow-up.	131
Correa S	2018	Cys-C	prospective	Increasing concentration of Cys-C was associated with a 28% higher hazard of cardiovascular death or heart failure hospitalization (HR:1.28, P<0.001).<br>Cys-C was associated with a higher hazard of CVD, myocardial infarction, or stroke (HR 1.15, P<0.01).	132
Shen G	2018	Cys-C	retrospective	As cystatin C levels from low to high(< 0.84, 0.84-1.03 and ≥ 1.04mg/L), all-cause mortalities were increased 0.9%, 3.7% and 9.5% (P < 0.001), as well as the composite endpoints, 11.1%, 21.7% and 40.7%, respectively (P < 0.001).	133
Akgul O	2013	Cys-C	prospective	Higher in-hospital and 1-month cardiovascular mortality rates were observed in the Cys-C>1.12 mg/L group (9.4% vs. 1.6%, P<0.001 and 14.5% vs. 2.2%, P<0.001, respectively).<br>Admission Cys-C >1.12 mg/L was a independent predictor of one-month cardiovascular mortality (odds ratio, 5.3; P=0.02).	134

Devaux Y	2012	miRNA-208b miRNA-499	unknown	miRNA-208b and miR-499 were increased in MI patients (>10(5)-fold, P < 0.001);In patients who presented <3 h after onset of pain, miR-499 was positive in 93% of patients and hs-cTnT in 88% of patients (P= 0.78);miR-499 and hs-cTnT provided comparable diagnostic value with areas under the ROC curves of 0.97.	136
D'Alessandra Y	2010	miRNAs	unknown	Acute MI up-regulated miRNA-1, -133a, -133b, and -499-5p plasma levels, both in humans and mice, whereas miR-122 and miRNA-375 were lower than control only in STEMI patients.	137
Adachi T	2010	miRNA-499	unknown	miR-499 was produced almost exclusively in the heart. Plasma miR-499 concentrations were measurably increased in all individuals with AMI	138
Kuwabara Y	2011	miRNAs	unknown	Mouse model of MI indicated that the levels of miR-1, miR-133a, miR-208a, and miR-499 were significantly reduced in the infarcted myocardium. Circulating miR-133a in patients with cardiovascular diseases originate mainly from the injured myocardium.	141
Li M	2018	lncRNAs	unknown	The ROC curve showed that LIPCAR (AUC=0.782) had better diagnostic accuracy. higher levels of LIPCAR were independent predictors of major adverse cardiovascular events in patients with STEMI (HR=5.93, P=0.001).	142
Gao L	2017	lncRNAs	cohort study animal model	HOTAIR expression was decreased in the serum of AMI patients and in mice subjected to coronary artery ligation. The adenovirus vector-driven overexpression of HOTAIR limited hypoxia-induced myocyte apoptosis.	143
Kumarswamy R	2014	LIPCAR	prospective	LIPCAR was downregulated early after MI but upregulated during later stages. LIPCAR levels identified patients developing cardiac remodeling and were independently to other risk markers associated with future cardiovascular deaths.	145
Zhao P	2018	lncRNAs	prospective	Compared to the lncRNA expression profiles of noncoronary artery controls, a total of 106 differentially expressed lncRNAs were discriminated in AMI patients, including 40 upregulated lncRNAs and 66 downregulated lncRNAs (P < 0.05).	146
Hu Y	2015	SIRT1	unknown	The mRNA expression levels of the SIRT1 gene in the microarray study were significantly lower in the AMI,UA and overall ACS patients(all p<0.01);There is a significant differences in the SIRT1 mRNA expression among the allelic genes of rs3758391 (p < 0.01) in the healthy participants.	148
Akkafa F	2015	SIRT1	unknown	SIRT1 expression were reduced in both HF subtypes, particularly in dHF. (p=0.002, control vs cHF; p<0.001, control vs dHF). SIRT1 expression was correlated with the oxidant levels and antioxidant capacity.	149
Duarte VHR	2019	TREM4	unknown	TREM4 is upregulated in the early phase of ACS. Increased TREM4 mRNA expression in blood leukocytes is influenced by gene polymorphisms. TREM4 polymorphisms were not associated with coronary lesion extent.	150
Silbiger VN	2013	whole genomic expression analysis	unknown	A total of 549 genes were found to be differentially expressed in the first 48 h after the ACS-Ph1.	151
Boufenzer A	2015	TREM-1	prospective	TREM-1 expression is upregulated in ischemic myocardium in mice and humans. TREM-1 blockade ameliorates cardiac function and limits ventricular remodeling	152
Schopfer DW	2014	GDF-15	prospective	Each doubling in GDF-15 was associated with a 2.5-fold increased rate of CV events (HR:2.53, P <0.001). Participants GDF-15>2660 ng/L had higher mortality compared with those GDF-15<1770 ng/L (HR 2.73, P ≤0.001). Addition of GDF-15 to existing risk factors resulted in a 50% change in net reclassification of patients' risk for mortality.	154
Wang X	2016	GDF-15	unknown	Serum GDF-15 levels were increased in CAD grou(p< 0.001). A positive correlation was observed between GDF-15 and the Gensini score (r = 0.85, P < 0.001). Serum GDF-15 level had a 80.0% sensitivity and 91.7% specificity for predicting CAD.	155

Gutiérrez-Leonar	2017	PAPP-A<br>CAT<br>SOD-1<br>SOD-2	unknown	Coronary PAPP-A levels were elevated among patients at risk for cardiovascular disease(p< 0.05); Antioxidant enzyme activities were higher in coronary samples than in peripheral samples from subjects with ischemic cardiopathy secondary to atherosclerosis (P < 0.001).	160
Iversen KK	2008	PAPP-A<br>CKMB<br>troponin T	unknown	Mean PAPP-A values at admission were higher in patients with STEMI(p <0.01);In samples drawn <2 hours after admission, the sensitivity of PAPP-A was superior (93%) to that of CKMB (60%) and troponin T (61%).	161
Lund J	2003	cTn<br>PAPP-A<br>CRP	retrospective	At a cutoff level of 2.9 mIU/L, elevated PAPP-A was an independent predictor of adverse outcome (RR:4.6, P=0.002). Another independent predictor was admission CRP >2.0 mg/L (RR, 2.6; P=0.03).	162
Tan B	2019	PRMT5	unknown	Expression levels of the PRMT5 gene in peripheral blood from patients with AMI are lower than in patients with stable cardiovascular disease (Z=-4.813,p=0.000).Patients who have a low PRMT5 expression in the peripheral blood are 5.472 times more likely to suffer from AMI than other patients.	164
Tan B	2019	PIK3C2A	retrospective	PIK3C2A gene expression in peripheral blood of AMI patients was lower than one in the non-coronary heart disease subjects. Low expression of PIK3C2A gene was an independent risk factor of AMI and increased the risk of AMI by 2.231 folds.	165

BNP: B-type natriuretic peptide; CAD: coronary artery disease; CAT: catalase; CD62P: platelet expression of P-selectin; cHF: compensated heart failure; CysC: cystatin C; ECG: electrocardiogram; eGFR: estimated glomerular filtration rate; ESM-1: endothelial cell-specific molecule 1; eNOS: endothelial nitric oxide synthase; Gal-3: galectin-3 GDF-15: growth-differentiation factor-15: h-FABP: heart-type fatty acid binding protein; HFpEF: preserved left ventricular ejection fraction; HOTAIR: HOX antisense intergenic RNA; IL-33: interleukin-33; IL-37: interleukin-37; LAVi: LA volume index; LIPCAR: mitochondrial long noncoding RNA uc022bqs.1; lncRNA: long noncoding RNA; LPLT: large platelet; Lp-PLA2: lipoprotein-associated phospholipase A2; LVEF: left ventricular ejection fraction; MACE: major adverse cardiovascular events; mi-RNA: microRNA; MPV: mean platelet volume; MPV/P: mean platelet volume to platelet count ratio; MPV/PC: mean platelet volume/platelet count; MRV: mitral regurgitant volume; PAPP-A: Pregnancy-Associated Plasma Protein-A; PIK3C2A: class II phosphatidylinositol 3-phosphate kinase; PLT: platelet; pmiRNAs: platelet microRNAs; PPMT5: protein arginine methyltransferase 5; PRMT5: the protein arginine methyltransferase 5; RVSP: right ventricular systolic pressure; SAP: stable angina pectoris; sCD40L: soluble CD40 ligand; SIRT-1: sirtuin 1; sICAM-1: soluble intercellular adhesion molecule-1; SOD-1: superoxide dismutase-1; SOD-2: superoxide dismutase-2; sST2: soluble suppression of tumorigenicity 2; ST2: suppression of tumorigenicity 2; suPAR: soluble urokinase plasminogen activator receptor; SVCAM-1: soluble vascular cell adhesion molecule-a; TREM-1: triggering receptor expressed on myeloid cells 1; TREM-4: triggering receptor expressed on myeloid cells 4; UAP: unstable angina pectoris; VEGF: vascular endothelial growth factor; β-TG: beta-thromboglobulin
